# Vagal nerve magnetic stimulation for post-traumatic cricopharyngeal achalasia with bilateral vocal cord paralysis: A case report

**DOI:** 10.1097/MD.0000000000043525

**Published:** 2025-07-25

**Authors:** Lirong Liu, Shujuan Huang, Hanbo Chen, Si Chen, Jinfeng Liang, Churong Liu

**Affiliations:** a Department of Rehabilitation, Guang Dong Sanjiu Brain Hospital, Guangzhou, Guangdong Province, China.

**Keywords:** bilateral vocal cord paralysis, cricopharyngeal achalasia, neurorehabilitation, post-traumatic dysphagia, vagus nerve magnetic stimulation

## Abstract

**Rationale:**

Post-traumatic dysphagia is a severe complication of traumatic brain injury, particularly in cases involving medullary damage. The rare combination of cricopharyngeal achalasia and bilateral vocal cord paralysis presents substantial challenges, with profound swallowing dysfunction and increased aspiration risk. Current treatments primarily target cricopharyngeal dysfunction but lack effective solutions for concurrent vocal cord paralysis, highlighting the need for innovative therapeutic strategies.

**Patient concerns:**

A 15-year-old male presented with severe dysphagia, confirmed as upper esophageal sphincter (UES) dysfunction and bilateral vocal cord paralysis. The patient exhibited severe aspiration (grade V water swallowing test, penetration aspiration scale: score of 7) and elevated UES residual pressure (28.2 mm Hg, normal < 12.0 mm Hg).

**Diagnoses:**

Post-traumatic neurogenic dysphagia with cricopharyngeal achalasia and bilateral vocal cord paralysis secondary to medullary damage.

**Interventions:**

An individualized protocol combining vagus nerve magnetic stimulation (VNMS) and conventional rehabilitation was implemented. VNMS targeted the left vagus nerve using 5 Hz stimulation at 80% resting motor threshold, while rehabilitation included pulmonary exercises, balloon dilation therapy, and tongue base pressure training.

**Outcomes:**

Substantial improvements were observed. UES residual pressure decreased from 28.2 mm Hg to 2.7 mm Hg, penetration aspiration scale score improved from 7 to 2, and the functional oral intake scale increased from level 1 to level 6, enabling oral feeding and removal of the tracheostomy and gastric tube. No adverse events were reported.

**Lessons:**

This case highlights the potential of VNMS as a noninvasive and effective treatment for complex post-traumatic brain injury dysphagia involving cricopharyngeal achalasia and bilateral vocal cord paralysis. By addressing dual swallowing-related pathologies, VNMS offers a promising therapeutic approach in neurorehabilitation. Further research is warranted to validate these findings and explore broader clinical applications.

## 1. Introduction

Post-traumatic dysphagia is a severe and complex complication following traumatic brain injury (TBI), particularly when medullary damage is involved. This condition may present as the rare combination of cricopharyngeal achalasia and bilateral vocal cord paralysis, leading to profound swallowing dysfunction, increased risk of aspiration, and significantly reduced quality of life.^[1,2]^ Post-traumatic dysphagia, like other TBI-related complications such as hydrocephalus, underscores the need for innovative therapeutic approaches, with recent advances highlighting the potential of neuromodulation and other strategies.^[3]^ Current conventional therapeutic approaches, including botulinum toxin injection, surgical myotomy, and neuromuscular electrical stimulation, primarily target cricopharyngeal dysfunction but lack effective interventions for concurrent vocal cord paralysis. This limitation underscores the urgent need for innovative therapeutic strategies to address such complex cases.^[4]^

In recent years, neuromodulation techniques have demonstrated significant potential in the field of neurorehabilitation. Vagus nerve stimulation (VNS) and transcranial magnetic stimulation (TMS) have shown promising results in promoting neuroplasticity and functional recovery in various neurological disorders.^[5,6]^ Among these, vagal nerve magnetic stimulation (VNMS), a novel noninvasive neuromodulation technique, has exhibited neuroprotective and functional recovery effects in conditions such as TBI and ischemic stroke.^[7–10]^ However, its potential role in addressing both cricopharyngeal dysfunction and vocal cord paralysis simultaneously has not yet been explored.

This case report documents the first application of VNMS in the treatment of post-traumatic dysphagia with concurrent cricopharyngeal dysfunction and bilateral vocal cord paralysis. Three key findings are highlighted: (1) the simultaneous improvement of dual swallowing-related pathologies through VNMS; (2) significant functional recovery validated by high-resolution manometry and laryngoscopy; and (3) evidence supporting the safety, feasibility, and therapeutic potential of VNMS in a complex case refractory to conventional treatments. By addressing these dual pathologies, this study provides preliminary evidence supporting neuromodulation as a transformative approach in neurorehabilitation.

## 2. Case presentation

### 2.1. Patient information

A previously healthy 15-year-old male sustained severe TBI in a motor vehicle accident on October 14, 2023. Emergency decompressive craniectomy was performed at a local hospital, followed by intensive care unit management, including tracheostomy and gastric tube placement to support respiration and nutrition. The patient was transferred to our hospital for the first time on October 28, 2023. During the comprehensive rehabilitation process, the patient gradually regained consciousness, reaching full consciousness on November 25, 2023, and successfully underwent cranioplasty on January 12, 2024. Despite 2 courses of comprehensive inpatient rehabilitation (comprising anti-infection therapy, neurotrophic medication, pulmonary rehabilitation, balloon dilation training, and direct current induction electrical stimulation) the patient was admitted to our rehabilitation department for the third time on March 13, 2024, with persistent severe neurogenic dysphagia.

**Figure 1.**
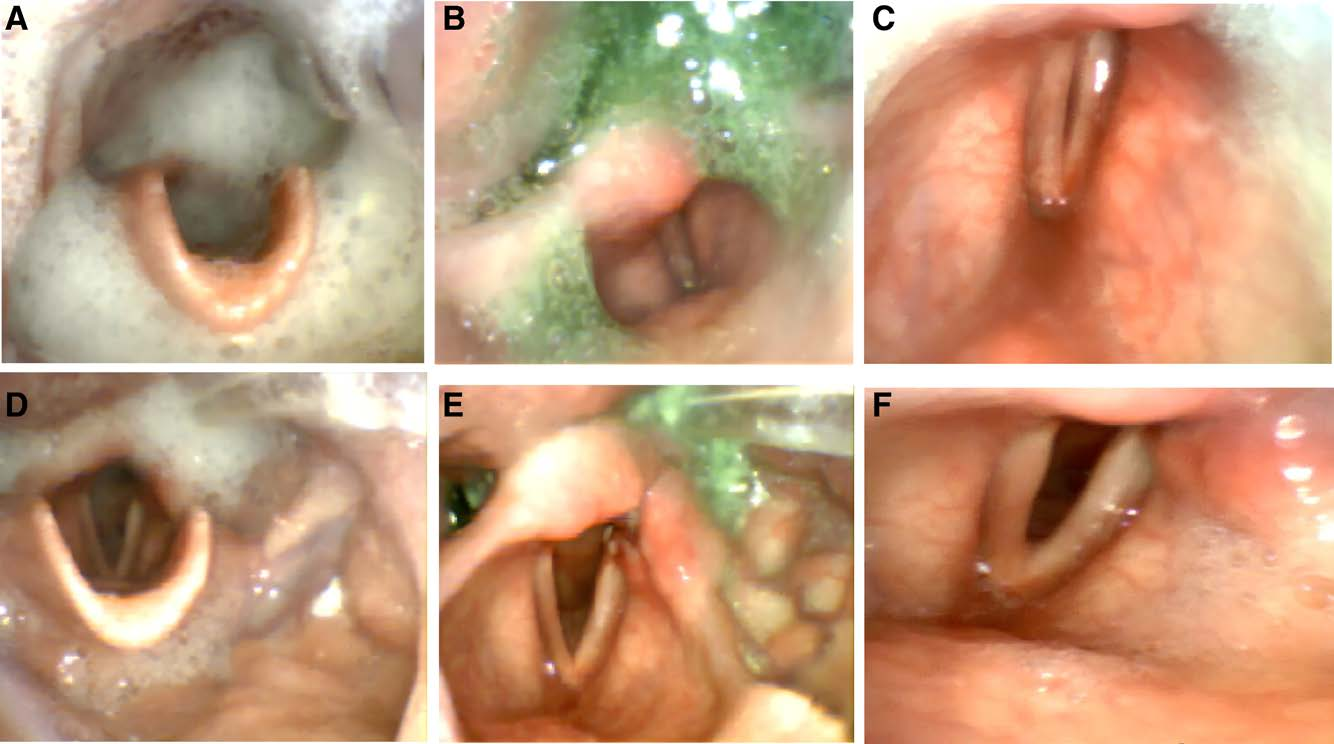
(A–C) Before treatment, FEES demonstrated severe penetration/aspiration, significant pharyngeal residue, and bilateral vocal cord paralysis with inability to abduct and adduct. (D–F) After treatment, FEES showed reduced penetration/aspiration, markedly decreased pharyngeal residue, and improved bilateral vocal cord mobility compared to the pretreatment state.

**Figure 2.**
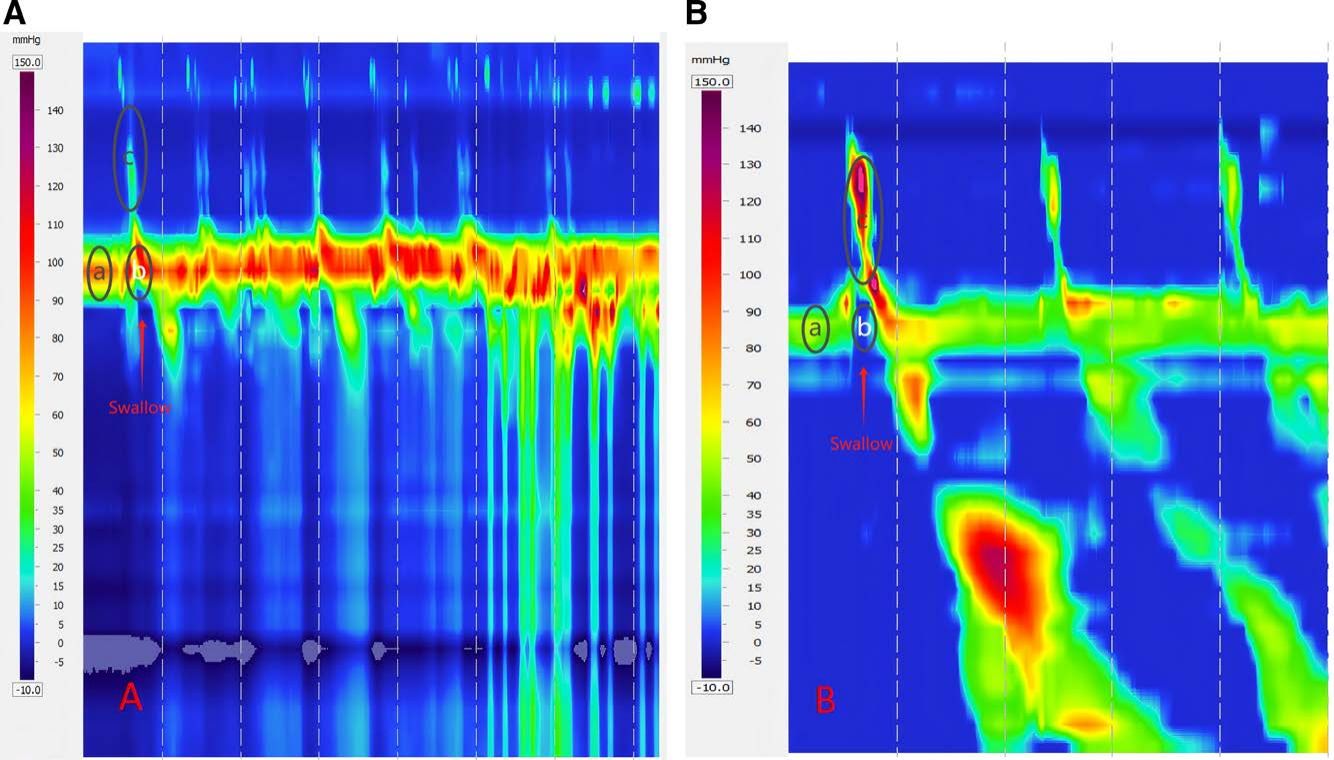
(A) Before treatment, HRM revealed poor UES relaxation (indicated by elevated UES resting pressure at point “a” and increased post-swallow UES relaxation residual pressure at point “b”), weak pharyngeal contraction (shown by reduced peak pharyngeal contraction pressure at point “c”), and poor coordination between UES relaxation and pharyngeal contraction (cricopharyngeal achalasia - hypertensive type). (B) After treatment, HRM demonstrated improved UES relaxation (normalized UES resting pressure at point “a” and decreased post-swallow UES relaxation residual pressure at point “b”), enhanced pharyngeal contraction (increased peak pharyngeal contraction pressure at point “c”), and adequate coordination between UES relaxation and pharyngeal contraction. HRM = high-resolution manometry, UES = upper esophageal sphincter.

Brain imaging revealed low-density lesions in the medulla and the left frontotemporoparietal region. Clinically, the patient exhibited hallmark symptoms of medullary paralysis, including absent pharyngeal reflex, poor cough reflex, and a significant risk of aspiration. Objective swallowing assessments confirmed severe dysfunction: grade V modified water swallowing test (severe aspiration), a penetration aspiration scale (PAS) score of 7 (indicating a high risk of aspiration) (Fig. 1A; Table 1), and elevated upper esophageal sphincter (UES) residual pressure of 28.2 mm Hg (normal < 12.0 mm Hg) with cricopharyngeal achalasia on high-resolution manometry (Fig. 2A; Table 2). Fiberoptic endoscopic evaluation revealed bilateral vocal cord paralysis in a neutral position (Fig. 1B and C; Table 1).

**Table 1:** Swallowing function parameters before and after treatment.

Parameters	Before treatment	After treatment	Reference ranges
Vocal cord adduction	Left: fixed Right: fixed	Left: normal Right: normal	Left: normal Right: normal
Vocal cord abduction	Left: fixed Right: fixed	Left: weakened Right: weakened	Left: normal Right: normal
Murray scale (0–3 scores)	3	1	0 scores
Yale scale (1–5 grade)	5	2	1 grade
PAS scale (1–8 grade)	7	2	1 grade
Modified water swallowing test (5 mL: I–V)	V	II	II
Functional oral intake scale (level 1–7)	Level 1	Level 6	Level 7

PAS = penetration aspiration scale.

**Table 2:** Changes in UES regional parameters before and after treatment.

UES parameters	Before treatment	After treatment	Reference ranges
Resting pressure (mm Hg)	53.6	37.2	34–104
Residual pressure (mm Hg)	28.2	2.7	<12.0
Time to relaxation nadir (ms)	18	230	74–365
Relaxation duration (ms)	43	506	480–1020
Recovery time (ms)	25	276	259–760

UES = upper esophageal sphincter.

**Figure 3.**
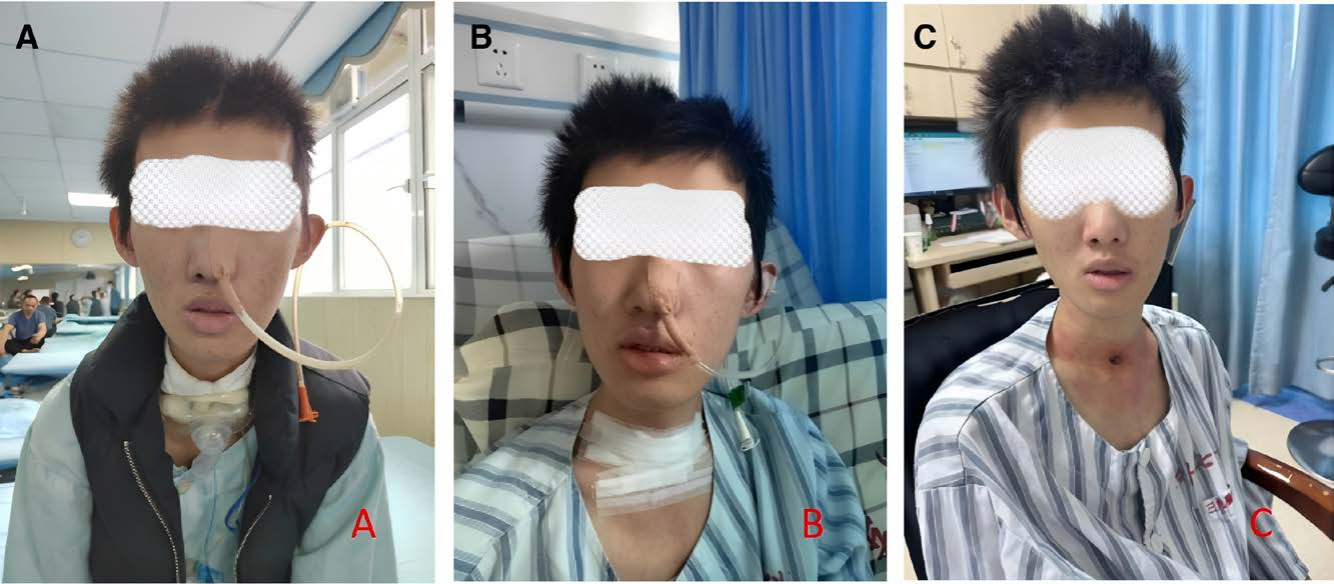
(A) Before treatment (March 22, 2024), the patient had indwelling tracheostomy tube and nasogastric tube. (B) After treatment (April 15, 2024), the tracheostomy tube was successfully decannulated. (C) After treatment (April 18, 2024), the nasogastric tube was successfully removed.

The patient had no significant past medical history, and his family history was unremarkable for neurological or swallowing disorders. Despite prior conventional rehabilitation interventions, the patient remained completely dependent on an artificial airway and enteral feeding (Fig. 3A). The primary therapeutic goals were defined as the removal of the tracheostomy and gastric tubes and the restoration of safe oral intake.

### 2.2. Diagnostic assessment

**Figure 4.**
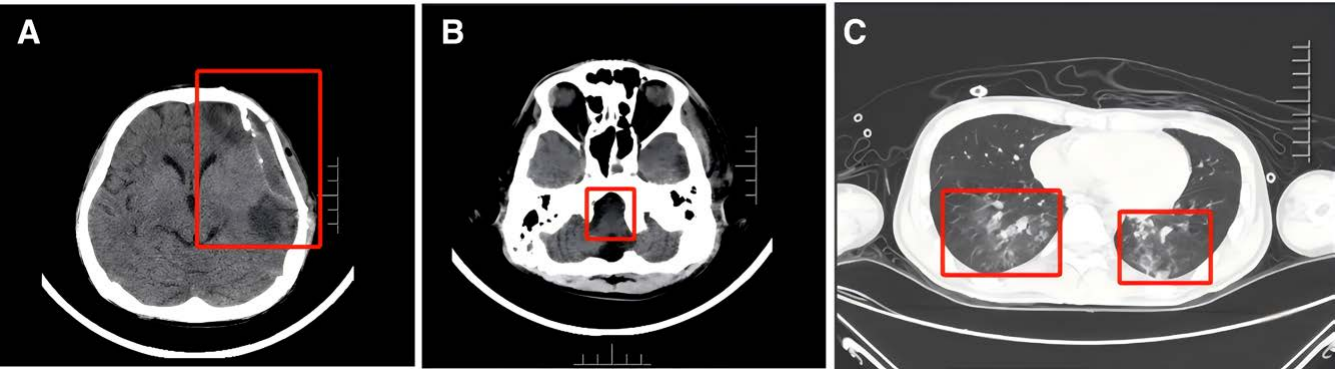
(A and B) CT of the brain demonstrated low-density lesions in the medulla and left frontotemporoparietal region. (C) CT of chest showed multiple inflammatory patchy shadows in both lungs.

The initial neurological examination revealed that the patient was alert and oriented but exhibited medullary nerve dysfunction, manifesting as severe swallowing and laryngeal impairments. A head CT performed on March 14, 2024, identified softening lesions in the left frontotemporal-parietal lobe and medulla oblongata, with resolution of paranasal sinusitis and mastoid inflammation (Fig. 4A and B).

Comprehensive swallowing function assessments demonstrated severe impairment. The modified water swallowing test was classified as grade V (severe aspiration), and PAS score was 7, indicating a high risk of aspiration. Fiberoptic laryngoscopy revealed bilateral vocal cord fixation in a neutral position (unable to abduct or adduct), with a Murray score of grade 3 and a Yale Pharyngeal Residue Severity Rating Scale score of grade 5 (Fig. 1A–C; Table 1). High-resolution manometry indicated severe UES dysfunction, characterized by elevated residual pressure (28.2 mm Hg; normal < 12.0 mm Hg), shortened relaxation duration (43 ms; normal 480–1020 ms), and poor coordination between UES relaxation and pharyngeal contraction (Fig. 2A; Table 2). These findings confirmed pronounced cricopharyngeal achalasia, which contributed to the patient’s severe dysphagia and aspiration risk.

Based on these comprehensive evaluations, the patient was diagnosed with severe post-traumatic neurogenic dysphagia, characterized by cricopharyngeal achalasia and bilateral vocal cord paralysis, secondary to medullary dysfunction following traumatic brain injury. The prognosis indicated a high risk for aspiration pneumonia (Fig. 4C), complete dependence on artificial feeding, an anticipated extended rehabilitation period, and recovery potential influenced by the extent of medullary involvement.

### 2.3. Therapeutic intervention

Based on the comprehensive assessment of severe neurogenic dysphagia with cricopharyngeal achalasia and bilateral vocal cord paralysis, an individualized treatment protocol was developed. It should be noted that the patient had previously undergone 2 courses of conventional rehabilitation with limited improvement in swallowing function, necessitating the exploration of additional therapeutic approaches. This protocol combined conventional rehabilitation, VNMS and comprehensive safety monitoring. The primary therapeutic goals were to improve swallowing function, reduce aspiration risk, and enhance the patient’s overall quality of life.

The conventional rehabilitation program was continued and included pulmonary rehabilitation and swallowing function training. Pulmonary rehabilitation involved daily 15-minute breathing exercises, progressive voice training, and gradual tracheostomy tube capping based on patient tolerance. Swallowing function training consisted of balloon dilation therapy (5 sessions per week), tongue base pressure training (15 repetitions daily), and speaking valve adaptation exercises to improve vocal cord mobility and airway protection. These interventions were designed to address the mechanical and functional impairments contributing to dysphagia and aspiration risk.

**Figure 5.**
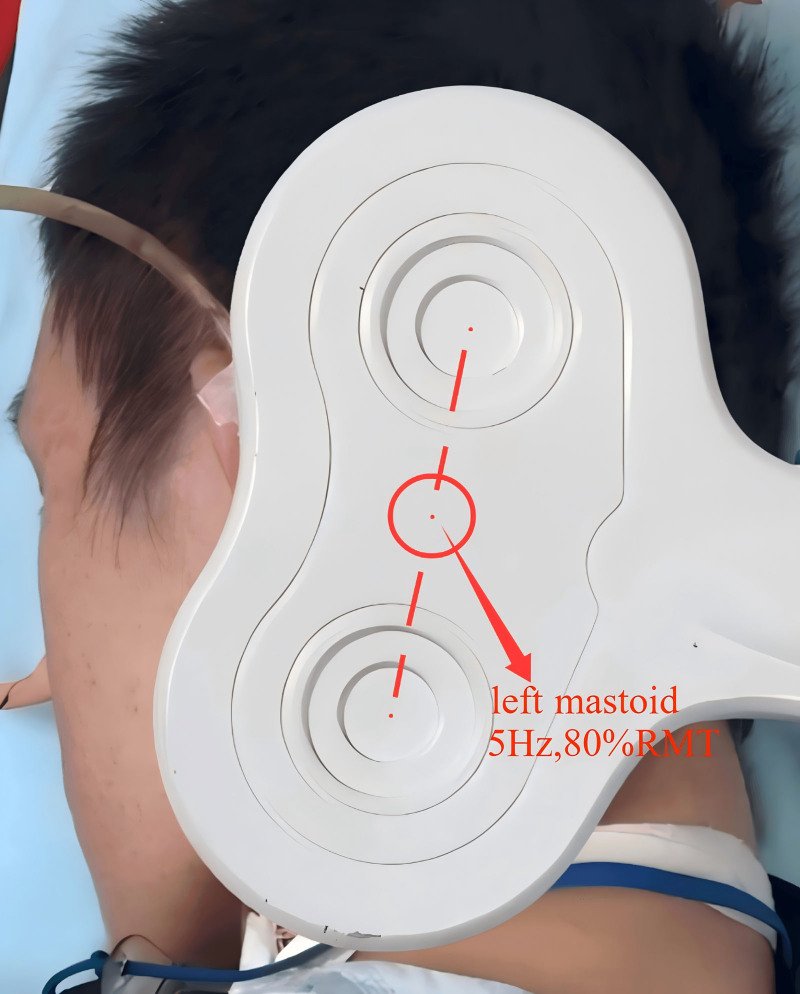
The stimulation coil was centered precisely over the left mastoid process to stimulate the proximal segment of the vagus nerve as it exits the jugular foramen, using stimulation parameters of 5 Hz and 80% RMT. RMT = resting motor threshold.

In addition to the conventional rehabilitation program, VNMS was introduced as part of the treatment plan starting on March 22, 2024. VNMS was performed using a YDR CCY-I transcranial magnetic stimulator (Yiruide, Wuhan, China) equipped with a figure-8 coil (Fig. 5). To minimize potential cardiac complications (as the right vagus nerve traverses the sinoatrial node and may affect heart rate^[7]^), the left vagus nerve was targeted. During the procedure, the patient was positioned in the lateral decubitus position, and the coil was precisely placed at the left mastoid process to stimulate the proximal segment of the vagus nerve as it exits the jugular foramen. The stimulation protocol consisted of 5 Hz frequency pulses delivered at 80% of the resting motor threshold (RMT), with 6-second stimulation periods followed by 24-second intervals, delivering a total of 1200 pulses per session over 20 minutes. The selection of 5 Hz stimulation frequency was based on electrophysiological evidence indicating that frequencies in the 5 to 10 Hz range optimally modulate vagal nerve activity to influence swallowing-related neural circuits.^[7]^ The 80% RMT intensity was chosen based on established safety protocols for TMS and precedent studies demonstrating both efficacy and safety at this intensity level for dysphagia rehabilitation.^[11,12]^ This parameter combination has been successfully employed in prior investigations of vagus nerve magnetic stimulation for poststroke dysphagia.^[7]^ RMT was determined using single-pulse stimulation and surface electrodes by identifying the minimum stimulation intensity required to elicit motor-evoked potentials (MEP) or minimal muscle movement in relaxed target muscles, such as the suprahyoid muscle group.^[12]^

Safety monitoring and treatment evaluation were integral components of the protocol to ensure patient safety and optimize therapeutic outcomes. Regular assessments included swallowing function tests (modified water swallowing test, PAS score), fiberoptic endoscopic examination, high-resolution manometry, and patient feedback. During the treatment period, the patient did not experience any significant adverse reactions, such as vagal nerve overstimulation or local discomfort, confirming the safety and tolerability of the protocol. The treatment plan was dynamically adjusted based on the patient’s progress, leading to significant improvements in swallowing function and a marked reduction in aspiration risk.

### 2.4. Follow-up and outcomes

During the follow-up period from March 22 to April 18, 2024, comprehensive evaluations demonstrated significant improvements across multiple functional parameters. A telephone follow-up at 6 months posttreatment confirmed that the patient maintained normal swallowing function with regular oral intake of food and liquids, without any adverse effects. UES measurements showed notable normalization, with resting pressure improving from 53.6 to 37.2 mm Hg (normal range: 34–104 mm Hg), residual pressure decreasing from 28.2 to 2.7 mm Hg (target: <12.0 mm Hg), time to relaxation nadir improving from 18 to 230 ms (normal range: 74–365 ms), relaxation duration increasing from 43 to 506 ms (normal range: 480–1020 ms), and recovery time improving from 25 to 276 ms (normal range: 259–760 ms) (Fig. 2A and B; Table 2).

Swallowing function assessments revealed substantial progress. The PAS score improved from 7 to 2, the Murray Scale advanced from grade 3 to 1, the Yale Pharyngeal Residue Severity Rating improved from grade 5 to 2, and the functional oral intake scale (FOIS) progressed from level 1 (complete dependence on artificial feeding) to level 6 (oral feeding without special preparation). Laryngoscopic examination revealed varying degrees of recovery in bilateral vocal cord adduction and abduction functions (Fig. 1A–F; Table 1). The vocal cord abduction dysfunction was primarily attributed to injury of the recurrent laryngeal branch of the vagus nerve or medullary damage.^[13,14]^ Vocal cord function plays a critical role in breathing, influencing the coordination of breathing and swallowing,^[15]^ as well as airway protection during forced expiration or coughing, which is essential for tracheostomy tube removal.^[16]^

These functional improvements enabled 2 major clinical milestones: successful tracheostomy tube removal on April 15 (Fig. 3B) and gastric tube removal on April 18 (Fig. 3C), allowing the patient to transition to complete oral feeding. Throughout the treatment period, no adverse events were reported, and the patient demonstrated excellent compliance. A long-term follow-up protocol was established, including regular swallowing assessments, periodic laryngoscopic evaluations, and continued rehabilitation exercises to maintain and further improve functional gains.

## 3. Discussion

This case highlights the therapeutic potential of VNMS combined with conventional rehabilitation in the management of complex neurogenic dysphagia, specifically post-TBI dysphagia with cricopharyngeal achalasia and bilateral vocal cord paralysis. Several notable features of this case underscore the efficacy of this approach. First, the concurrent presence of cricopharyngeal achalasia and bilateral vocal cord paralysis, objectively documented through high-resolution manometry and laryngoscopy, presents a unique and challenging clinical scenario. Second,notable improvements were observed across multiple quantitative parameters, including a reduction in UES residual pressure (from 28.2–2.7 mm Hg), an increase in relaxation duration (from 43–506 ms), and enhancements in swallowing function assessments such as PAS scores (from 7–2) and FOIS progression (from level 1–level 6). These findings indicate the potential of VNMS to address multiple aspects of swallowing dysfunction simultaneously.

The vagus nerve and its branches play a pivotal role in swallowing through distinct innervation patterns. The superior laryngeal nerve provides sensory innervation to the valleculae and structures above the vocal folds, while its external branch supplies motor innervation to the cricothyroid muscle. The recurrent laryngeal nerve, another branch of the vagus nerve, innervates all intrinsic laryngeal muscles except the cricothyroid muscle, including the cricopharyngeal muscle, and provides sensory input from the mucosa below the vocal folds. VNMS, by targeting the proximal segment of the left vagus nerve at the mastoid process, simultaneously activates these 2 critical branches, creating dual physiological pathways: on one hand, it enhances cricothyroid muscle function through the external branch of the superior laryngeal nerve, improving vocal cord tension and adduction capability; on the other hand, it enhances intrinsic laryngeal muscles and cricopharyngeal muscle function through the recurrent laryngeal nerve, improving vocal cord abduction and cricopharyngeal relaxation. This synchronized activation of both pathways explains the concurrent improvements in cricopharyngeal function and vocal cord mobility observed in our case.

Additionally, VNMS may enhance the excitability of the vagal nuclei (located in the medulla), strengthening the central pattern generator (CPG) function responsible for coordinating the activity of swallowing and respiration-related muscle groups.^[7,17]^ This central modulatory effect further promotes the synergistic function of laryngeal and pharyngeal muscles. Previous studies have shown that vagus nerve stimulation can induce laryngeal MEPs, which serve as biomarkers for effective nerve activation, supporting this central regulatory mechanism.^[18]^

VNMS likely exerts its therapeutic effects by enhancing the function of swallowing-related muscles, such as the cricopharyngeal muscle and vocal cords, through targeted stimulation of these vagal branches. This anatomical understanding aligns with previous findings on the efficacy of vagus nerve stimulation in improving swallowing and vocal cord function.^[15,19]^

When comparing VNMS with other neuromodulation techniques, several distinct advantages and limitations can be identified.^[8]^ Unlike electrical vagus nerve stimulation, which requires surgical implantation of electrodes and a pulse generator, VNMS offers a completely noninvasive approach, eliminating surgical risks and complications while maintaining the ability to directly modulate vagal pathways.^[18]^ This noninvasive nature makes VNMS particularly suitable for initial therapeutic trials before considering more invasive interventions.^[20]^ Compared to TMS, which primarily targets cortical regions controlling swallowing function, VNMS directly modulates the peripheral nerve pathways involved in both UES function and vocal cord movement, potentially offering more precise targeting of the dual pathologies observed in our case. Transcranial magnetic stimulation primarily enhances cortical excitability and promotes neuroplasticity in swallowing-related motor cortex areas but has limited direct effect on laryngeal function compared to VNMS.^[21]^ These comparative insights highlight the potential role of VNMS as a valuable addition to the neuromodulation toolkit, particularly for cases involving both cricopharyngeal dysfunction and vocal cord paralysis.

Neuromodulation techniques, including VNMS, improve swallowing function through multiple mechanisms. Lin et al^[7]^ demonstrated that VNMS significantly improved swallowing function in patients with brainstem stroke, as evidenced by increased CP-MEP amplitude and shortened latency. These findings suggest that peripheral nerve stimulation may enhance the function of the CPG and promote neuroplasticity. Similarly, Zhang et al^[5]^ revealed that vagus nerve stimulation enhances post-TBI neural function by modulating neuroinflammation and promoting neuroplasticity. VNMS’s potential to enhance neuroplasticity aligns with other regenerative therapies, such as stem cell therapy for dementia, which have shown promise in promoting neural recovery.^[22]^ Neuromodulation techniques like VNMS may promote neuroplasticity, as seen in other neurological conditions such as epilepsy, where molecular mechanisms underlying neural recovery have been explored. This cross-disease perspective reinforces the understanding that neural recovery may follow similar pathways across different neurological disorders.^[23]^ This neuroplasticity mechanism shares similarities with regenerative approaches such as stem cell therapy, which has shown promise in promoting neural recovery through similar pathways of neuroregeneration and neuroplasticity in various neurological disorders.^[24]^ Vespa et al^[18]^ further supported the role of VNS-induced laryngeal motor evoked potentials as biomarkers for effective nerve activation. In our case, these mechanisms likely contributed to the comprehensive functional recovery observed, including improvements in both UES function and vocal cord mobility.

This case is particularly significant as it demonstrates simultaneous improvements in UES function and vocal cord mobility, 2 critical components of swallowing and airway protection. The use of multiple assessment tools, including high-resolution manometry, laryngoscopy, and validated swallowing function scales, provides robust evidence for the efficacy of VNMS. Furthermore, the observed improvements across various functional parameters suggest that VNMS may offer a comprehensive therapeutic approach for complex neurogenic dysphagia. Importantly, VNMS represents a less invasive alternative to surgical interventions such as cricopharyngeal myotomy,^[25]^ potentially reducing the risks associated with surgery while providing comparable functional benefits.

Despite these promising results, certain limitations must be acknowledged. As a single case report, the generalizability of our findings is inherently limited. Larger-scale randomized controlled trials are needed to validate the therapeutic efficacy and safety of VNMS across diverse patient populations and etiologies of neurogenic dysphagia. Additionally, further research is warranted to optimize stimulation parameters and explore the long-term effects of VNMS on swallowing function and quality of life. The successful application of VNMS in this case underscores the need for equitable access to advanced neurorehabilitation therapies, particularly for underserved populations with severe neurological conditions.^[26]^

## 4. Conclusions

This case report demonstrates that VNMS combined with conventional rehabilitation therapy could be an effective treatment option for complex post-TBI dysphagia involving both cricopharyngeal achalasia and bilateral vocal cord paralysis. The significant improvements observed in multiple objective parameters, including UES residual pressure, relaxation duration, PAS scores, and FOIS levels, highlight the therapeutic potential of this approach. These findings align with prior research on the role of neuromodulation in promoting neuroplasticity, enhancing CPG function, and improving swallowing-related muscle coordination. Importantly, VNMS offers a noninvasive alternative to surgical interventions, such as cricopharyngeal myotomy, potentially reducing associated risks while achieving comparable functional outcomes. It is important to note, however, that this case report has several limitations. First, due to the combined intervention design, we are unable to definitively attribute the observed improvements solely to VNMS. The patient had undergone 2 courses of comprehensive conventional rehabilitation prior to the initiation of VNMS with limited improvement in swallowing and vocal cord function, suggesting that VNMS may have provided an incremental benefit. Nevertheless, the continued application of conventional rehabilitation techniques may have created favorable conditions for recovery, and synergistic effects between the 2 interventions cannot be ruled out, as suggested by previous studies on combined neuromodulation and conventional therapies. However, as a single case report, the generalizability of these findings is limited. To establish causality and validate the therapeutic effects observed in this study, well-designed randomized controlled trials (RCTs) with adequate sample sizes are essential. Such trials should include control groups receiving either sham stimulation or conventional rehabilitation alone to isolate the specific contribution of VNMS. Additionally, multicenter RCTs encompassing diverse etiologies of neurogenic dysphagia, varying injury severities, and different time windows post-injury will be crucial for determining the generalizability of VNMS and optimizing patient selection. Larger-scale randomized controlled trials are necessary to confirm the efficacy and safety of VNMS across diverse patient populations and etiologies of neurogenic dysphagia. Additionally, further research is warranted to optimize stimulation parameters and explore the long-term effects of VNMS on swallowing function and quality of life. Despite these limitations, this case provides preliminary evidence that VNMS may serve as a promising therapeutic option for managing severe neurogenic dysphagia, particularly in cases where multiple swallowing-related structures are affected.

## Acknowledgments

We would like to express our gratitude to the staff at the Department of Rehabilitation, Guang Dong Sanjiu Brain Hospital, for their valuable advice and assistance. We also acknowledge the use of the Generative AI tool, SiderAI, and are deeply indebted to the subject who participated in the study for his consent and cooperation.

## Author contributions

**Conceptualization:** Lirong Liu, Shujuan Huang, Hanbo Chen, Churong Liu.

**Data curation:** Shujuan Huang.

**Formal analysis:** Lirong Liu, Shujuan Huang, Hanbo Chen.

**Investigation:** Lirong Liu, Shujuan Huang, Si Chen.

**Methodology:** Lirong Liu, Shujuan Huang, Hanbo Chen, Jinfeng Liang.

**Project administration:** Shujuan Huang.

**Resources:** Lirong Liu, Hanbo Chen.

**Software:** Hanbo Chen.

**Supervision:** Lirong Liu, Hanbo Chen, Churong Liu.

**Validation:** Hanbo Chen.

**Visualization:** Si Chen.

**Writing – original draft:** Lirong Liu, Shujuan Huang.

**Writing – review & editing:** Lirong Liu, Shujuan Huang, Hanbo Chen, Si Chen, Jinfeng Liang, Churong Liu.

## References

[1] Sugi T Kanazawa H Takinami A. A case of post-trauma dysphagia: peculiar swallowing dynamics due to associated laryngeal paralysis. Prog Rehabil Med. 2020;5:20200003. 32789271 10.2490/prm.20200003 PMC7365201

[2] Kawasaki S Giagka V de Haas M. Pressure measurement of geometrically curved ultrasound transducer array for spatially specific stimulation of the vagus nerve. 2019 9th International IEEE/EMBS Conference on Neural Engineering(NER). 2019:1239-1242.

[3] Sanker V Kundu M El Kassem S. Posttraumatic hydrocephalus: recent advances and new therapeutic strategies. Health Sci Rep. 2023;6:e1713. 38028696 10.1002/hsr2.1713 PMC10652704

[4] Evancho A Tyler WJ McGregor K. A review of combined neuromodulation and physical therapy interventions for enhanced neurorehabilitation. Front Hum Neurosci. 2023;17:1151218. 37545593 10.3389/fnhum.2023.1151218 PMC10400781

[5] Zhang H Li CL Qu Y Yang Y-X Du J Zhao Y. Effects and neuroprotective mechanisms of vagus nerve stimulation on cognitive impairment with traumatic brain injury in animal studies: a systematic review and meta-analysis. Front Neurol. 2022;13:963334. 36237612 10.3389/fneur.2022.963334 PMC9551312

[6] Florie M Pilz W Dijkman RH. The effect of cranial nerve stimulation on swallowing: a systematic review. Dysphagia. 2021;36:216–30. 32410202 10.1007/s00455-020-10126-x PMC8004503

[7] Lin WS Chou CL Chang MH Chung Y-M Lin F-G Tsai P-Y. Vagus nerve magnetic modulation facilitates dysphagia recovery in patients with stroke involving the brainstem – a proof of concept study. Brain Stimul. 2018;11:264–70. 29162502 10.1016/j.brs.2017.10.021

[8] Ylikoski J Lehtimäki J Pirvola U. Non-invasive vagus nerve stimulation reduces sympathetic preponderance in patients with tinnitus. Acta Otolaryngol. 2017;137:426–31. 28084177 10.1080/00016489.2016.1269197

[9] Hays SA Rennaker RL Kilgard MP. Targeting plasticity with vagus nerve stimulation to treat neurological disease. Prog Brain Res. 2013;207:275–99. 24309259 10.1016/B978-0-444-63327-9.00010-2 PMC4615598

[10] Li L Wang D Pan H. Non-invasive vagus nerve stimulation in cerebral stroke: current status and future perspectives. Front Neurosci. 2022;16:820665. 35250458 10.3389/fnins.2022.820665 PMC8888683

[11] Wang L Wu Q Yang Z. Preliminary study of vagus nerve magnetic modulation in patients with prolonged disorders of consciousness. Neuropsychiatr Dis Treat. 2022;18:2171–9. 36187561 10.2147/NDT.S381681 PMC9522480

[12] Macrae PR Jones RD Huckabee ML. The effect of swallowing treatments on corticobulbar excitability: a review of transcranial magnetic stimulation induced motor evoked potentials. J Neurosci Methods. 2014;233:89–98. 24932964 10.1016/j.jneumeth.2014.06.010

[13] DeLozier KR Gould FDH Ohlemacher J Thexton AJ German RZ. Impact of recurrent laryngeal nerve lesion on oropharyngeal muscle activity and sensorimotor integration in an infant pig model. J Appl Physiol (1985). 2018;125:159–66. 29648522 10.1152/japplphysiol.00963.2017 PMC6086969

[14] Erman AB Kejner AE Hogikyan ND Feldman EL. Disorders of cranial nerves IX and X. Semin Neurol. 2009;29:85–92. 19214937 10.1055/s-0028-1124027 PMC4239699

[15] Gould FDH Lammers AR Mayerl CJ German RZ. Specific vagus nerve lesion have distinctive physiologic mechanisms of dysphagia. Front Neurol. 2019;10:1301. 31920925 10.3389/fneur.2019.01301 PMC6920241

[16] Wallace S McGrath BA. Laryngeal complications after tracheal intubation and tracheostomy. BJA Educ. 2021;21:250–7. 34178381 10.1016/j.bjae.2021.02.005 PMC8212164

[17] Bautista TG Sun QJ Pilowsky PM. The generation of pharyngeal phase of swallow and its coordination with breathing: interaction between the swallow and respiratory central pattern generators. Prog Brain Res. 2014;212:253–75. 25194202 10.1016/B978-0-444-63488-7.00013-6

[18] Vespa S Stumpp L Bouckaert C. Vagus nerve stimulation-induced laryngeal motor evoked potentials: a possible biomarker of effective nerve activation. Front Neurosci. 2019;13:880. 31507360 10.3389/fnins.2019.00880 PMC6718640

[19] Kumai Y Samejima Y Yumoto E. Postdeglutitive residue in vagus nerve paralysis and its association with feeding style. Eur Arch Otorhinolaryngol. 2016;273:4369–75. 27363405 10.1007/s00405-016-4182-3

[20] Redgrave J Day D Leung H. Safety and tolerability of transcutaneous vagus nerve stimulation in humans; a systematic review. Brain Stimul. 2018;11:1225–38. 30217648 10.1016/j.brs.2018.08.010

[21] Momosaki R Abo M Kakuda W. Bilateral repetitive transcranial magnetic stimulation combined with intensive swallowing rehabilitation for chronic stroke Dysphagia: a case series study. Case Rep Neurol. 2014;6:60–7. 24803904 10.1159/000360936 PMC4000294

[22] Uwishema O Ghezzawi M Wojtara M Esene IN Obamiro K. Stem cell therapy use in patients with dementia: a systematic review. Int J Emerg Med. 2025;18:95. 40350434 10.1186/s12245-025-00876-6 PMC12067764

[23] Shariff S Nouh HA Inshutiyimana S. Advances in understanding the pathogenesis of epilepsy: unraveling the molecular mechanisms: a cross-sectional study. Health Sci Rep. 2024;7:e1896. 38361811 10.1002/hsr2.1896 PMC10867297

[24] Pradhan AU Uwishema O Onyeaka H Adanur I Dost B. A review of stem cell therapy: an emerging treatment for dementia in Alzheimer’s and Parkinson’s disease. Brain Behav. 2022;12:e2740. 35971625 10.1002/brb3.2740 PMC9480940

[25] Regan J Murphy A Chiang M McMahon BP Coughlan T Walshe M. Botulinum toxin for upper oesophageal sphincter dysfunction in neurological swallowing disorders. Cochrane Database Syst Rev. 2014;2014:CD009968. 24801118 10.1002/14651858.CD009968.pub2 PMC10600350

[26] Uwishema O Boon P. Bridging the gaps: addressing inequities in neurological care for underserved populations. Eur J Neurol. 2025;32:e70073. 39912252 10.1111/ene.70073 PMC11799841

